# Expiration of State Licensure Waivers and Out-of-State Telemedicine Relationships

**DOI:** 10.1001/jamanetworkopen.2023.43697

**Published:** 2023-11-15

**Authors:** Eric Bressman, Rachel M. Werner, Daniel Cullen, Benjamin Ukert, Benjamin A. Barsky, Jennifer L. Kowalski, Ateev Mehrotra

**Affiliations:** 1 Department of Medicine, University of Pennsylvania School of Medicine, Philadelphia; 2 Leonard Davis Institute of Health Economics, University of Pennsylvania, Philadelphia; 3 Corporal Michael J. Crescenz Veterans Affairs Medical Center, Philadelphia, Pennsylvania; 4 Elevance Health, Indianapolis, Indiana; 5 Texas A&M University, College Station; 6 Harvard University, Cambridge, Massachusetts; 7 Department of Health Care Policy, Harvard Medical School, Boston, Massachusetts; 8 Division of General Medicine and Primary Care, Beth Israel Deaconess Medical Center, Boston, Massachusetts

## Abstract

This cross-sectional study compares the use of telemedicine in states where COVID-19 pandemic–related licensure waivers expired vs states where waivers continued.

## Introduction

Physicians generally must hold a license recognized in the state in which their patients are located.^1^ At the COVID-19 pandemic’s onset, almost all states implemented temporary licensure waivers, which allowed patients to obtain care from out-of-state clinicians via telemedicine.^2,3^ Over the course of the pandemic, most waivers expired.^4^ To inform the ongoing debate about reforming physician licensure to facilitate telemedicine, we compared out-of-state telemedicine relationships in states where licensure waivers expired vs states where waivers continued.

## Methods

Using Elevance Health claims data from January 2019 to June 2022, we identified patients living in 3 states where licensure waivers expired in mid-2021 (Colorado, Maine, Wisconsin) and 5 states where waivers continued at least through June 2022 (California, Georgia, Indiana, New Hampshire, New York). Patients without at least 90% of days covered in 2020 to 2022 were excluded. The University of Pennsylvania Institutional Review Board deemed this cross-sectional study exempt from review and informed consent because deidentified data were used. We followed the STROBE reporting guideline.

Out-of-state telemedicine relationships were defined as unique patient-clinician pairings with at least 2 visits, 1 of which was via telemedicine, in the preperiod (March 2020 to April 2021, when all states had active waivers). In the postperiod (July 2021 to June 2022, when waiver status varied by state), for each relationship, we captured whether there were any visits (primary outcome) and any telemedicine or in-person visits specifically (secondary outcomes).

Using multivariate logistic regression where the unit of analysis was out-of-state relationship, we estimated the association between the outcomes and state waiver status, adjusting for patient demographic (age, sex, race and ethnicity), geographic (rural, urban, suburban; distance to clinician), and relationship (number and type of preperiod visits, prepandemic relationships, known licensure in patients’ state) characteristics. We conducted similar regressions for subgroups of relationships, including clinician specialty, number of preperiod visits, distance apart, and state licensure status (eAppendix in Supplement 1 for details).

Two-sided *P* < .05 indicated statistical significance. Statistical analyses were performed with Stata 15.1 (StataCorp LLC).

## Results

During the preperiod, we identified 45 087 unique patients (27 554 females [61.1%], 17 533 males [38.9%]; mean [SD] age, 39.8 [18.9] years) and 55 845 out-of-state telemedicine relationships (Table). In states with expired vs continued waivers, more patients were from rural areas (14.7% vs 10.3%; *P* < .001) and patient-clinician distances were greater (>804.7 km: 59.5% vs 40.1%; *P* < .001).

**Table.** Characteristics of Out-of-State Telemedicine Relationships

Characteristic	No. (%)		*P* value
	States with continued licensure waiver	States with expired licensure waiver	
**Unique individuals**			
No. of patients	38 775	6312	
Age, mean (SD), y	39.8 (19.1)	40.2 (17.5)	.12
Sex^a^			
Male	15 280 (39.4)	2253 (35.7)	<.001
Female	23 495 (60.6)	4059 (64.3)	
Race and ethnicity^b^			
Asian	1647 (4.2)	143 (2.3)	<.001
Black	2345 (6.0)	104 (1.6)	
Hispanic	1695 (4.4)	108 (1.7)	
Native American and Pacific Islander	184 (0.5)	42 (0.7)	
White	28 398 (73.2)	5328 (84.4)	
Other race, multiracial, and unknown^c^	4506 (11.6)	587 (9.3)	
Location			
Rural	3992 (10.3)	931 (14.7)	<.001
Suburban	6267 (16.2)	1025 (16.2)	
Urban	28 023 (72.3)	4298 (68.1)	
Unknown	493 (1.3)	58 (0.9)	
**Unique relationships**			
No. of out-of-state telemedicine relationships	48 061	7784	
Distance between patient and clinician, km			
0-32.2	7538 (15.7)	879 (11.3)	<.001
>20-96.6	8906 (18.5)	538 (6.9)	
>96.6-193.1	3616 (7.5)	578 (7.4)	
>193.1-321.9	2646 (5.5)	382 (4.9)	
>321.9-804.7	4690 (9.8)	638 (8.2)	
>804.7	19 277 (40.1)	4635 (59.5)	
Unknown	1388 (2.9)	134 (1.7)	
No. of preperiod visits, mean (SD)			
Telemedicine	2.4 (2.6)	3.3 (3.9)	<.001
In-person	1.2 (1.8)	1.0 (1.8)	<.001
Overall	3.7 (3.1)	4.4 (4.1)	<.001
Pre–COVID-19 pandemic visit in 2019			
Yes	19 228 (40.0)	3323 (42.7)	<.001
No or unknown	28 833 (60.0)	4461 (57.3)	
Licensure in patients’ state			
Yes	3476 (7.2)	262 (3.4)	<.001
No or unknown	44 585 (92.8)	7522 (96.6)	

^a^ As reported in the data source.

^b^ Race and ethnicity data were obtained from the data source. Categories were mutually exclusive. Hispanic was included in the data source as an option for race.

^c^ Other race was included as an option in the data source.

In states with expired waivers, out-of-state relationships were less likely to have any postperiod visits (adjusted odds ratio [AOR], 0.76; 95% CI, 0.72-0.80) (Figure). The AOR was 0.65 (95% CI, 0.60-0.71) among relationships with 4 or more preperiod visits and was 0.65 (95% CI, 0.61-0.71) for those with patient-clinician distance over 321.9 km. There was no differential receipt of visits when the out-of-state clinician held a license in the patient’s state and among relationships with mental health practitioners. Out-of-state relationships were less likely to have both telemedicine (AOR, 0.90; 95% CI, 0.84-0.96) and in-person (AOR, 0.73; 95% CI, 0.68-0.77) visits after waiver expiration.

**Figure.**
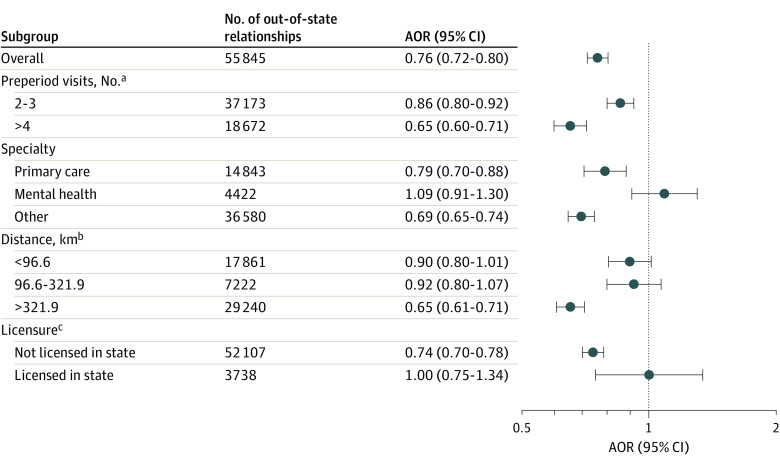
Association Between State Waiver Status (Expired vs Active) and Likelihood of Any Visits Between Patient and Clinician in the Year After Waiver Expiration Models were adjusted for patient demographic, geographic, and relationship characteristics. Error bars represent 95% CIs. AOR indicates adjusted odds ratio. ^a^Preperiod (period in which all states in the analysis had active licensure waivers) spanned March 2020 to April 2021. ^b^Distance between patient and clinician was measured between the centroid of their respective zip codes. ^c^Licensure was ascertained from publicly available, National Provider Identifier–linked data.

## Discussion

Out-of-state telemedicine visits surged while licensure waivers were active.^3,5^ This study highlighted the harms to access to care when the waivers expired. When the waivers expired, patients did not switch from telemedicine to in-person care but rather tended to stop seeing the physician altogether. This finding was most evident when the patient and clinician were over 321.9 km apart.

The results support the need to reform state licensure.^6^ Among the small number of telemedicine relationships in which out-of-state physicians held a license in states where patients resided, there was no decrease in continuity.

When waivers expired, out-of-state telemedicine did not simply stop. This finding may be attributed to many clinicians being unaware of the changes in state regulations. This study was limited by the sample of states with accessible data, the differences in their populations, and the possibility for residual confounding.

## Notes

Supplement 1.
eMethods.

Supplement 2.
Data Sharing Statement
