# Supplementary material for: Expiration of State Licensure Waivers and Out-of-State Telemedicine Relationships
Source: JAMA Netw Open. 2023 Nov 15;6(11):e2343697. doi: 10.1001/jamanetworkopen.2023.43697 (PMC10652155; doi:10.1001/jamanetworkopen.2023.43697)
Supplement: Supplement 1. — eMethods. [file jamanetwopen-e2343697-s001.pdf]

Supplemental Online Content

Bressman E, Werner RM, Cullen D, et al. Expiration of state licensure waivers and out-of-state telemedicine relationships. *JAMA Netw Open*. 2023;6(11):e2343697. doi:10.1001/jamanetworkopen.2023.43697

eMethods.

This supplemental material has been provided by the authors to give readers additional information about their work.

© 2023 Bressman E et al. *JAMA Network Open*

eMethods

Data Source
Our data included commercially insured individuals enrolled in an Elevance Health affiliated plan. Elevance Health's companies serve approximately 118 million people through a diverse portfolio of medical, digital, pharmacy, behavioral, clinical, and complex care solutions.

Visit Characteristics
Telemedicine visits were identified as outpatient evaluation and management (E&M) visits with any of the following telemedicine codes: place of service code (02, 10), modifier codes (GT, GQ, GO, FQ, 93, 95), or telemedicine-specific CPT codes (G0071, G2010, G2012, G2025, G2250-2, G2061-3, 99441-3, 98966–8, 99421-3, 99453, 99454, 99457, 99091, 95250-1, 98970-2). Urgent care visits (as identified by place of service code 20) were excluded.

Patient Characteristics
We pulled information on age, self-reported sex, and self-reported race for all patients. Self-reported ethnicity was missing for a large proportion of patients, and therefore was not used. The patient's state of residence was determined by their home address. We additionally looked at whether they lived in a rural, suburban or urban area.

Relationship and Provider Characteristics
An out-of-state relationship was considered one where the patient's state of residence and the provider's state of practice were discordant. In order to take the most conservative view of what likely constituted an out-of-state visit, we considered the provider's location in one of two ways: 1) the zip code associated with the provider found in the claim file, 2) the practice location associated with the provider's NPI. If these were both discordant with the patient's state of residence, this was considered to be an out-of-state visit.

We focused our analysis on established relationships, which we defined as having at least two visits during the pre-period (March 2020-April 2021). To be considered a telemedicine relationship, at least one of these visits had to be via telemedicine. The pre-period was selected because waivers were broadly active in all 8 states during this time.

We calculated the distance between the patient and provider using the centroid of the patient's census block group and the provider's zip code. Because the provider may have had two distinct indicators for location, as noted above (the zip code found in the claim file or the NPI-linked zip code), we used the closest of these.

We additionally looked at features of an out-of-state relationship that we hypothesized would be predictive of its likelihood of continuing over time: a) the intensity of the relationship, characterized by the total number of encounters that took place during the pre-period; b) whether, in addition to telemedicine (which was a pre-requisite for inclusion), there were also in-person visits during the pre-period; c) whether there was a visit (in-person or telemedicine) in the 14 months prior to the pandemic (between January 2019 and February 2020; of note, this was limited in that some of the patients included in the sample may not have had coverage for most of 2019); and d) whether the provider was known to hold a license in the patient's state of

© 2023 Bressman E et al. *JAMA Network Open*

residence (State licensure information was pulled from the NPPES file. This information is also limited in that it may not always be kept up to date, and may not reflect which licenses were held at the exact time of the visit).

For providers, we pulled information on their specialty using CMS specialty codes contained in the claims file and, when these were missing, an NPI-linked taxonomy code.

Unit of Analysis and Outcomes

We considered as our primary unit of analysis the out-of-state patient-provider relationship that was active during the pre-period (while waivers were broadly active). Patients could have more than one of these out-of-state relationships.

The primary outcome was whether this relationship had any visits during the post-period (July 2021-June 2022). We additionally looked at whether they had either a telemedicine or in-person visit, specifically, during the post-period. The post-period was selected because waivers had expired in all 3 waiver expiration states and remained active in the 5 other states during this time.

Analysis

For our primary analysis, we used a logistic regression model to estimate the association between state waiver status and likelihood that a relationship continued (i.e. had any visits) during the post-period. We adjusted for patient demographics (age, sex, race), geographic characteristics (rural vs urban vs suburban, distance to provider), and relationship characteristics (number and type of pre-period visits, pre-pandemic relationship, known licensure in patient's state). In order to better visualize which patient and relationship characteristics were most associated with sensitivity to waiver expiration, we conducted subgroup analyses using a similar logistic regression model. These included stratifying by number of pre-period visits (aka relationship intensity), provider specialty, distance between patient and provider, and whether the provider was known to hold a license in the patient's state of residence. Finally, we used an identical logistic regression model to estimate the association between state waiver status and likelihood that a relationship continued to have telemedicine visits or in-person visits (each outcome analyzed separately) during the post-period.

All statistical analyses used Stata software, version 15.1 (StataCorp LLC).

© 2023 Bressman E et al. *JAMA Network Open*
