# Supplementary material for: Expiration of State Licensure Waivers and Out-of-State Telemedicine Relationships
Source: JAMA Netw Open. 2023 Nov 15;6(11):e2343697. doi: 10.1001/jamanetworkopen.2023.43697 (PMC10652155; doi:10.1001/jamanetworkopen.2023.43697)
Supplement: Supplement 2. — Data Sharing Statement [file jamanetwopen-e2343697-s002.pdf]

Data Sharing Statement

Bressman. Expiration of State Licensure Waivers and Out-of-State Telemedicine Relationships. *JAMA Netw Open*. Published November 15, 2023. doi:10.1001/jamanetworkopen.2023.43697

Data
Data available: No
